# Gender-based violence in the context of armed conflict in Northern Ethiopia

**DOI:** 10.1186/s13031-023-00563-4

**Published:** 2024-01-03

**Authors:** Desalew Salew Tewabe, Muluken Azage, Gizachew Yismaw Wubetu, Sisay Awoke Fenta, Mulugeta Dile Worke, Amanu Mekonen Asres, Wallelign Alemnew Getnet, Genet Gedamu Kassie, Yonatan Menber, Alemtsehay Mekonnen Munea, Taye Zeru, Selamawit Alemayehu Bekele, Sadiya Osman Abdulahi, Tigist Biru Adamne, Hiwot Debebe Belete, Belay Bezabih Beyene, Melkamu Abte, Tesfaye B Mersha, Abel Fekadu Dadi, Daniel A Enquobahrie, Souci M. Frissa, Yonas E. Geda

**Affiliations:** 1 Amhara Public Health Institute, Amhara Region, Bahir Dar, Ethiopia; 2 School of Public Health, College of Medicine and Health Sciences, Bahir Dar University, P.o.Box: 79, Bahir Dar, Ethiopia; 3 Emergency Response and Recovery Officer, Amhara Region, Bahir Dar, Ethiopia; 4 Department of Midwifery, College of Health Sciences, Debre Tabor University, Debre Tabor, Ethiopia; 5 Faculty of Social Science, Bahir Dar University, Bahir Dar, Ethiopia; 6 Institute of Public Health, College of Medicine and Health Sciences, University of Gondar, Gondar, Ethiopia; 7 Bureau of Women Children and Social Affairs, Amhara Region, Bahir Dar, Ethiopia; 8 Amhara Regional Health Bureau, Amhara Region, Bahir Dar, Ethiopia; 9 Cincinnati Children’s Hospital Medical Center, University of Cincinnati, Cincinnati, OH USA; 10 Menzies School of Health Research, Charles Darwin University, Casuarina, Australia; 11 Department of Epidemiology, School of Public Health, University of Washington, Seattle, WA USA; 12 Institute of Psychiatry, Psychology and Neuroscience, Health Service and Population Research Department, Centre for Global Mental Health, King’s College London, London, UK; 13 Department of Neurology and the Franke Barrow Global Neuroscience Education Center, Barrow Neurological Institute, Phoenix, AZ USA

**Keywords:** Sexual violence, Rape, Physical violence, Psychological violence, Armed conflict

## Abstract

**Background:**

Gender-based violence (GBV) particularly against women is unfortunately common during armed conflicts. No rigorous and comprehensive empirical work has documented the extent of GBV and its consequences that took place during the two years of devastating armed conflict in Northern Ethiopia. This study aims to assess GBV and its consequences in war-torn areas of northern Ethiopia.

**Methods:**

We used a qualitative method augmented by quantitative method to enroll research participants. We conducted in-depth interviews to characterize the lived experiences of GBV survivors. All interviews were conducted confidentially. The data were collected to the point of data saturation. All interviews were transcribed verbatim into local language, translated into English, and analyzed using a thematic analysis approach. We also used reports from healthcare facilities and conducted a descriptive analysis of the demographic characteristics of study participants.

**Results:**

One thousand one hundred seventy-seven persons reported GBV to healthcare providers. The qualitative study identified several forms of violence (sexual, physical, and psychological). Gang rape against women including minors as young as 14 years old girls was reported. Additionally, the perpetrators sexually violated women who were pregnant, and elderly women as old as 65 years, who took refuge in religious institutions. The perpetrators committed direct assaults on the body with items (e.g., burning the body with cigarette fire) or weapons, holding women and girls as captives, and deprivation of sleep and food. GBV survivors reported stigma, prejudice, suicide attempts, nightmares, and hopelessness. GBV survivors dealt with the traumatic stress by outmigration (leaving their residences), seeking care at healthcare facilities, self-isolation, being silent, dropping out of school, and seeking counseling.

**Conclusion:**

GBV survivors were subjected to multiple and compounding types of violence, with a wide range of adverse health consequences for survivors and their families. GBV survivors require multifaceted interventions including psychological, health, and economic support to rehabilitate them to lead a productive life.

## Background

Worldwide, armed conflicts continue to uproot millions of people from their homes every year. Modern-day armed conflicts and mass atrocities are being beamed by digital media in real-time, thereby bringing the devastating impact of war to households globally. At the end of 2020, 48 million people were displaced due to conflict and violence in 59 countries [1]. One of the highest levels of displacements was recorded in Sub-Saharan Africa (6.8 million) [1]. The impact of conflict and war is not limited to the violation of individual human rights [2, 3]. Armed conflict also has a devastating effect on the health of the affected population leading to high mortality and morbidity [3–5]. The impact is worse in vulnerable groups including children, women, the elderly, and those with disabilities [2, 4].

Gender-based violence is one of the tragic outcomes of armed conflicts, and the magnitude of GBV varies by countries [6–8]. The United Nations (UN) defines GBV as “any act of gender-based violence [perpetrated by the family, community, or State] that results in, or is likely to result in physical, sexual or psychological harm or suffering to women, including threats of such acts, coercion or arbitrary deprivation of liberty, whether occurring in public or private life” [9]. The World Health Organization (WHO), classifies GBV to consist of physical, sexual, psychological, and deprivation or neglect [10]. GBV has short-and long-term health consequences such as reproductive health problems, surgical problems, and psychological problems [11, 12].

On November 4 of 2020, a devastating armed conflict broke out in Northern Ethiopia and lasted for two years until a peace treaty was signed on November 3 of 2022 [13].

Here we provide a brief context to the conflict. Ethiopia is an ancient country that was not colonized in Africa and home of the oldest remains of Homo Sapiens i.e. Australopithecus Afarensis (Lucy) inhabited Ethiopia about 3 million years ago. Ethiopia is mentioned in holy books and has a long history [14, 15]. The Solomonic dynasty dominated the political system for several centuries until it was overthrown by young military officers (locally known as Derg) in 1974 [16, 17]. The military rule lasted till 1991, at which time an armed movement dominated by an ethnic group from the North took power until it was removed from power in 2018, by a popular youth movement [16]. The ruling group then retreated to its base in the North called Tigray. Following a 4 years tension between the central government and the former ruling group, a devastating war broke out on November 4 of 2020.

The war took place in the Amhara, Afar and Tigray regions of Ethiopia. The war led to devastating consequences [18]. The current study was carried out by the Amhara Public Health Insitute and Bahir Dar University in the Amhara region. Between July 2021 and December 2021, six Zones in the Amhara Region, namely South Gondar, North Gondar, Wag Hemra, North Wollo, South Wollo, and North Showa, were invaded by an armed group called Tigray People Liberation Front (TPLF) [19]. Large-scale displacement continues to be reported from conflict-affected areas in Amhara and Afar [19, 20]. The armed conflict has led to many being internally displaced from their homes, the mass traumatization of the population and the sexual violence against girls and women [18]. However, GBV in the context of armed conflict and its psychosocial consequences in the region has not yet been well-documented. Therefore, this study aims to explore lived experiences, and consequences of survivors of GBV in war-affected areas of northern Ethiopia.

## Methods

### Study settings

**Fig. 1:**
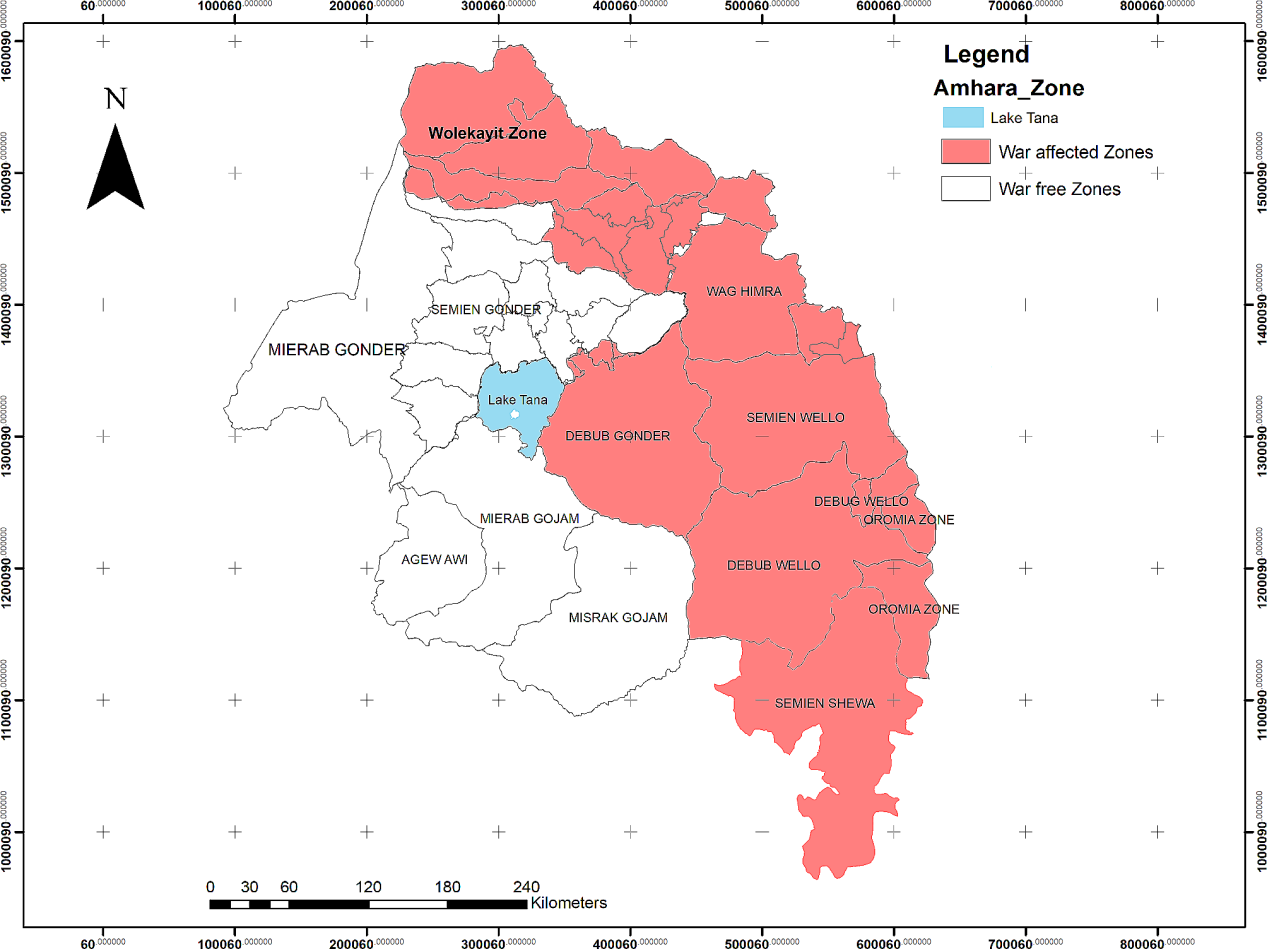
Map of War affected Zones in Amhara Region,Ethiopia

The study was conducted in Ethiopia, the second-most populous country in Africa, located in the horn of Africa region. Amhara Region is one of the eleven country’s regional states affected by the devastating war. The study was specifically carried out in seven war-affected zones and one city administration of the Amhara Region: North Wollo (Semien Wollo), South Wollo (Debub Wollo), North Gondar (Semien Gondar), South Gondar (Debub Gondar), Oromo, North Showa (Semien Showa), Wag Hemra Zones, and Dessie city administration (Fig. 1).

The estimated population of the war-affected zones in the Amhara Region is 11, 926, 815 and the total internal displaced people in war-affected areas at the time of writing of the manuscript was 6,142,944.

### Study design and population

Quantitative and qualitative methods were used to conduct the study in the war-affected areas of Amhara Region. All GBV survivors (n = 1177) who came to healthcare facility or gender office to seek a healthcare or help in war-affected areas were included to describe their sociodemographic characteristics. The study took place between December 24, 2021, and January 14, 2022. An in-depth interview guide was used to interview the survivors of GBV. Additional data were also acquired from key informant interviews to explore the extent of GBV and the pattern of health services utilization among GBV survivors.

The study employed snowball sampling techniques to enroll study participants in war-affected areas. Snowball sampling is a procedure of selecting study participants in the community based on their relevance to the research issues by identifying the first case and then tracing others through the first case [21]. The adequacy of the sample was determined based on data saturation.

### Data collection tools and procedure

Women with MPH level training in public health who had more than three years of qualitative data collection experience were trained to interview the GBV survivors using an in-depth interview (IDI) guide. Key informant interview (KII) was also conducted on health facility heads and representatives who are knowledgeable about the health service needs of facilities in war affected areas using KIIguide to explore the health needs of GBV survivors, and data extraction checklist was prepared to collect sociodemographic characteristics of GBV survivors.

**For Qualitative data**: The IDI and KII guides were prepared by reviewing literature on GBV or related issues and customized in to the local context and translated into Amharic languages then evaluated by qualitative experts and used to collect data. Before starting the qualitative data collection, the interviewers introduced themselves and clearly explained the purpose and benefits of the study, their participation is voluntary and then obtain Informed consent was taken from each study participant. Once their consent was obtained, each respondent arranged the interview date and place in advance. Those respondents who were ready to be interviewed on the first contact were interviewed on the same day. All interviews were conducted confidentially in a sensitive manner by respecting the respondent’s convenient place and time of interview to make the participant feel relaxed and comfortable for the interview whereas majority of them prefer special rooms in health facilities. The IDI and KII were held in Amharic. All study participants and researchers were fluent in Amharic, which is the local as well as the official language of Ethiopia. The key informant interviews lasted for about 40 min, while the IDI lasted from 1:00 h. to 1:30 h. The data were collected to the point of saturation. A total of 18KII and 15 IDI participated.

A data extraction checklist was also used to collect demographic variables such as sex, age, place of residence and date of violence of the GBV survivors in war-affected areas.

### Data quality assurance (trustworthiness)

Triangulation, iterative questioning, member checking, and peer debriefing were made to ensure credibility. To triangulate the data, the study included different groups of participants, including GBV survivors and healthcare facility heads/representatives to explore the health needs of GBV survivors and healthcare services received. The data collectors were familiar with the cultural and social norms of the study participants and the study area. Member checking sessions were organized to present the preliminary findings of the data. As to transferability, a thick description was used to show that the research findings can be applied to other contexts, circumstances, and situations. Dependability was achieved using overlapping methods, i.e., an in-depth and key informant interview. Furthermore, using a tape recorder, careful probing, and interviewing activities were used to ensure dependability [22].

### Data analysis

Quantitative data were analyzed by the interviewers and the principal investigator descriptively. All interviews were carefully transcribed verbatim into Amharic (local language) without losing contextual meaning, translated into English, and analyzed using thematic analysis. The analysis involved the following steps: data familiarization, coding, identification of a thematic framework, indexing, charting and data interpretation. Data familiarization occurred through the processes of reading the transcriptions several times. This was followed by the extraction of meaning units from the transcripts. The meaning units were condensed by shortening the original text while maintaining the central meaning. The condensed versions were later assigned codes grouped into similar categories. We used both inductive and deductive approaches to account for categories that were known a priori and those that originated from the data.

### Researcher reflexivity

Reflexivity was maintained based on the recommended methodology [23, 24]. We acknowledge that the face-to-face interaction with participants might have been influenced by the researcher’s background, experience, prior assumptions, and values. This may potentially shape the conversation and impact the participant’s willingness to talk openly. The data acquisition was carried out by study personnel with practical experiences in interviewing gender-based violence survivors using an in-depth interview guide.

On the other hand, the research team constituted of professionals from various disciplines, including reproductive health, public health, behavioral science, psychiatry, behavioral neurology, and mental health professionals who had research and practice-based experiences on the topic of traumatic stress. The emerging themes and interpretations were extensively discussed among co-authors. Hence, the interpretation draws from the combined insights of those working on the data closely and members of the team with a wider perspective of methodology. The fact that the researchers come from different disciplinary backgrounds helped in making a balanced interpretation of the data.

The interview was conducted by women study staff members who share the same cultural background as the participants to capture the survivor’s lived experience in phenomenological way. The co-authors are of Ethiopian descent. Seventeen of the twenty-two authors live and work in the study area and the rest live abroad in US and UK. The research team had regular discussions to ensure that they are guided by their collective cultural knowledge.

### Ethics approval

The Institutional Review Board (IRB) of Amhara Public Health Institute reviewed the proposal of the study and provided ethical approval. Verbal informed consent to participate in the study was obtained from participants and their parents or legal guardian for those participants aged under 18 years. Verbal informed consent is acceptable and approved by the IRB of the institute.

## Results

One thousand one hundred seventy-seven survivors GBV survivors reported their trauma to healthcare facilities in war-affected areas. Six of the GBV survivors are men. The highest proportion was in North Wollo (39.0%), whereas the lowest was in the Oromo special Zone (1.7%). Of the total GBV survivors, 9.3% were aged below 18 years (Table 1).

**Table 1:** Number of SGBV survivors in the war-affected area from June 2021 to December 2021, north Ethiopia. (See Fig. 1 for the map of the regions stated below)

Zones	< 18 years, n (%)	≥ 18 years, n(%)	Frequency	Percent
North Wollo	33	426	459	39.0
South Wollo	30	204	234	19.9
North Showa	15	83	98	8.3
Wag Hemra	6	90	96	8.2
Dessie city	9	80	89	7.6
South Gondar	0	89	89	7.6
North Gondar	12	45	57	4.8
Kombolcha town	0	35	35	3.0
Oromo special zone	5	15	20	1.7
Total	**110**	**1067**	**1177**	**100**

### Qualitative findings

Four prominent themes (sexual violence, physical violence, psychological violence, and coping strategies) emerged from the data (Table 2).

**Table 2:** Sociodemographic characteristics of women exposed to sexual violence from June 2021 to December 2021 in Amhara region war-affected areas, Ethiopia

		Age		Total
		< 18 years	≥ 18 yrs.	
Victims’ residence	South Wollo		2	2
	North Gondar	4	2	6
	North Showa	1	2	3
	Wag Hemra		4	4
	Dessie city		1	1
Marital status	Single			10 6
	Married			

The four themes were found in all the transcripts. The guides allowed participants to express their feelings about the traumatic threat. The participants’ insights and perceived explanations about the trauma aided in developing the themes. Under each theme, the consequences of each violence, such as economic loss, mental health issues, physical injury, unwanted pregnancy, and other illnesses were identified. Similarly, each theme’s implications and impacts were explained.

### Sexual violence

#### Sexual violence as a means of political revenge

This theme explores the exposure of the respondents to sexual violence. Because of the breakdown of social infrastructure, the disintegration of families and communities, women and girls, including children were victims of sexual violence including rape and gang rape. The GBV survivors referred to the perpetrators as “Tigrayan invaders,” “Junta”, “TPLF”, “Woyane” (hereafter referred to as “perpetrators”) sexually violated girls as young as 14-year-old, pregnant women, and women as old as 65 years of age who were ill and who resided at religious places. Furthermore, the respondents described how the perpetrators raped the survivors in front of their family members (husbands, mothers, fathers, and children) and assaulted them using various deception mechanisms such as ordering them to fetch water and asking for help, threatening them by harming their relatives and firing a gun close to their head for intimidation and subjugation. Some rape survivors stated that they were pleading with the perpetrators to use condoms. Despite knowing that some of the victims were HIV/AIDS positive, the perpetrators refused to use condoms. The victims were also subjected to humiliating and demeaning verbal abuse such as “*you are Abiy’s (prime minister of Ethiopia) donkey”* and “*this is the smallest punishment for the Amhara ethnicity*.” Furthermore, some Tigrayan residents who were community members prior to the war were used as agents to identify wives and daughters of national and regional defense forces and political leaders of any position to be raped by invaders.

“[ …] then, two Tigray terrorist force members came into my house and told me that they were informed, as I am the wife of the federal police officer and Abiy Ahmed (prime minister of Ethiopia) supporter. They searched for the weapon but did not get anything in the house and went back. They came again in the evening, and they both raped me in front of my children [she was overwhelmed with grief and cried profusely].” (Source: a 35-year-old married woman).

A 32-year-old HIV/ADIS patient reported the following trauma:

“[…] they disrobed me by force. I was diagnosed positive for HIV/AIDS a year ago. Due to this, I informed and advised them to use condoms, but they did not believe me. All the four terrorist members raped me one by one.” (Source: a 32-year-old married woman).

Another Survivor added:

“[…] they forced and took my sister and me to the closed houses of our displaced neighbors. They raped both of us the whole night in separate rooms, and we came back to our home in the morning. I quietly escaped when the perpetrator fell asleep. My sister is 14 years old, and she lost her virginity and was sexually violated [profound sadness was noted].” (Source: a 30-year-old married women).

Similarly, girls and women detained for extended periods in detention houses set up by the perpetrators were viciously raped. Victims mentioned the elements of rape as:

“[…] other forms of coitus in awkward was forcefully practiced.” [Source: a 32-year-old married woman].

#### Stigma and ostracization

“I had been discriminated against too much. I usually worried and afraid in the village and did not go with anyone. They did not care for me even in church while they talked. So far, for the same reason, I have not visited my family and lived in a rented house at Debark.” (Source: 19 year-old married young woman who is in the tenth grade).

Another participant added:

“People in our surroundings remind us every day about the situation though we want to forget that. We feel comfortable after we come here. “Since we have a restaurant, customers always talk about it as if there is nothing to talk about and the ideas they talk about were out of the truth as we were interested in going with the invading soldiers.” (Source: an 18 year-old housemaid who is in the 11th grade).

#### Physical trauma

This theme focused on the physical trauma committed by the perpetrators. It is one of the most frequently reported events before, during, and after rape in a conflict situation. Armed combatants use various forms of physical trauma against females living in war-torn areas of the Amhara region, regardless of their age and pregnancy status. Slapping, hitting, hanging, biting, and burning the body with cigarette fire were the common physical trauma occurred among survivors.

##### Slapping

For most victims, slapping in the face was the most common form of physical violence in war-affected areas. In addition to the perpetrators’ interest in harming women and girls, they slapped their victims primarily to show their power and express their thoughts. Also, they used slapping as a means of warning the victims not to shout.

“[…] He [the armed man] forced me to enter the dark house to search for the weapons, but nothing was there, and I begged him to let me return to my home. However, he beat me with a stick, shoved me into the room, and put me on the mattress. Then I stood up and pleaded him by the name of an Ark, but he slapped me in the face and whacked me on the leg with a stick, so I fell and did what he wanted.” (Source: A 30-year-old married woman).

##### Hitting

Perpetrators used any material that could harm the women or girls who lived in the invaded areas. They hit victims until they were bloodied. They beat women and girls simply for any attempt to refuse the perpetrators, including refusals, to have sex with them. The perpetrators do not care if the victims were alive or dead. They beat every part of their bodies with a stick or stone.

“[…] However, he refused and hit me with a stick on my hand and leg, resulting in all my body being covered with blood.” (Source: a 30-year-old married woman)

“[…] Nevertheless, he ordered me to turn around and go far away from him, and if I did not, he warned as he could kill me. The child who was with me was shocked. Then he hit me with a stone and chased me.] (Source: An 18-year-old in the 11th grade).

##### Hanging and biting

The females in the invaded areas did not have the right to ask for their dignity. They recounted that, if they even tried to shout for help or project any emotional response to the pain they encountered, they would face severe consequences from their perpetrators. Every one of the perpetrators could practice what they want. There was no responsible person to punish them for their evil activities. They hung women and girls for any reason. Some of the females were also bitten.

“[…] the man was following us and came again to us. He hung me and said, “Aren’t you a woman? Am I not a man?” I said as I am a woman, and he is a man. In addition, he said, “Don’t I have a right to kiss you?” I told him that he could not rule over anyone but himself. He slapped me and said, “Who are you, and you do not talk to me?” (Source: an 18 year-old girl in the 11th grade).

“It was the first time I had been raped, so I shouted because I was so sick, and when I shouted, he hung me and covered my mouth with his hat, and he threatened me, saying he would kill me. Even in the morning, he was not interested in letting me go to my home and beat me.” (Source: a 14 year-old in the 8th grade).

##### Burning the body with cigarette fire

The perpetrators were smoking cigarettes until it was their turn to have sex. When one of them had sex with a woman, the other member used cigarette fire to harm the woman by burning her skin on the abdomen till it was wounded and left with a scar.

“[…].All of them raped me turn by turn, repeatedly. Other forms of coitus were forcefully practiced through every opening in the body. They smoked a cigarette and burnt my body (stomach) with the cigarette fire while they raped me. Here are the marks [showing the scars]” (Source: a 32 year-old married woman).

#### Post-traumatic pain

Victims suffered from post-traumatic pain due to physical trauma. The victims were exposed to different physical injuries, including loss of consciousness, bleeding, lasting scars on their bodies and back pain. This all has its implication on the life of the victims by lowering self-esteem, economic loss, and social discrimination:

“I was sick of the attack and suffering from the pain. (Source: a 14 years old 8th -grade student). I feel pain on my back. Still there is a wound, and there is bad odour.” (Source: a 32 year old married woman).

“Finally, I was unable to endure the suffering. […] He [the armed man] did what he wanted [rape]. Also, other group members were waiting to rape me again […] Then, after I tied the bleeding areas on my hand and leg, I left home through the door at the back of the house and went back to my home. Then I lost consciousness.” (Source: a 30 year old married woman).

Aside from the numerous physical and psychological consequences of violence against women, the impact on the community’s “social health” was negative and widespread. As victims became isolated by their families and communities, social bonds became negatively impacted.

#### Verbal Violence as a means of revenge

**Insults (individual, ethnic and political)**: The perpetrators used any means possible to humiliate Amhara people psychologically. Residents in war-torn areas were subjected to much psychological violence, including insults. To make the locals feel surrendered, the invading group insulted and intimidated them. Once they gained dominance in society, they increased acts of manipulation on community members. Surprisingly, the insults were systematized into individual, ethnic and political dimensions; more specifically, they attempted to depict the Amhara in animal terms by consistently stating: ‘You are Abiy Ahmed’s donkey’, which is intended to insult people of the Amhara as a whole. They also accuse the Amhara of having a long-term monopoly on the country’s politics. They do so in order to instill the wrong and baseless rhetoric on which they have been relying for decades to spread hatred against the Amhara:

“[….] While we were in Debre Berhan hospital for medication, we were served below standard service. On top of rape, the ‘Juntas’ (local name for perpetrators) have insulted and intimidated us, saying ‘you are Abiy’s donkey.” (Source: a 19 year old Student).

#### Stigma and discrimination

According to the survivors, the situation was bitter because the combatants and the community saw them negatively because of the violence they faced and treated them negatively because of the sexual and physical violence they experienced. Social stigma and discrimination exacerbated their victimisation and hampered their efforts to obtain the necessary assistance:

“I still came here for my illness but got it worse. The worst thing after I got harmed by the invaders was that people talked and each other and laughed at the situation, feeling embarrassed when I was passing by. Some people think that the situation occurred of our willingness that made me mad. Finally, I was made to think that all the people were talking about me and feel ashamed if they were talking about me. On the night of their release, I left Shoa Robit and headed for the countryside. Eight days after the Junta invaders left, the women and youth affair office women told us that if there was a victim of rape, “Come and tell me and you will be treated.” (Source: a 14 year old, 8th grade girl).

Another participant added:

“There is stigma and discrimination. Some say Junta (local name for perpetrators) raped you. Some whispered when they saw me. Some laughed and asked me, are you the raped one? Instead of caring and hesitating about the occasion, others are still negligent and do not care about my personality. I fear the public and prefer to die [feeling sad]. There is stigma and discrimination in the community. Our willingness does not do it, and there should be moral support to all the victims in the community.”(Source: a 32 year old married woman).

#### Fear and trauma

The survivors of GBV reported intrusive thoughts about the traumatic event, re-experiencing symptoms, recurring distress/anxiety, flashbacks, and avoidance of similar situations:

“[….] Ever since the incident, I prefer to be alone as I was afraid of the gossip and whispers against me. I could not sleep at night, and one of my relatives suggested I speak about the situation for treatment. I was praying for her health for fear of the infection. I did not want to tell others because I was afraid they might make fun of me.”(Source: a 32 year old woman who is an 8th grade).

Other participants added.

“I lost my confidence, and everything is boring to me. I bought more than 400,000 Birr, and now I hate this house, remembering the rape that happened to me at this house. Sometimes I want to shout. I feel pain in my back. Still, there is a wound, and there is a foul odor. […] Yes, there is stigma and discrimination. Some say Junta raped you. Some whispered when they saw me. Some laughed and asked me, are you the raped one? Instead of caring and hesitating about the occasion, others are still negligent and do not care about my personality. I fear the public and prefer to die [feeling sad].(Source: a 32 year old woman who is an 8th grade).

“The very painful thing is that my 14-year-old child learns that ‘Juntas’ raped his mother (me). However, I tried to keep the issue hidden to free him from psychological harm. He repeatedly asks me about what happened to me, which makes me worried. My neighbors ask my husband and my relatives about my well-being. I feel bad being the topic of discussion/gossip. This morning, when I was fetching water, people were staring at me at a distance as they heard what happened to me (crying).” (Source: a 30 year old married woman).

#### Hopelessness and low self-esteem

Long-term low self-esteem is especially debilitating when it impacts on the ability of victims’ ability to live a full and happy life. Respondents reported how their low self-esteem affected their careers, friendships, romantic relationships, and willingness to try new things. Despite the passage of time, their negative self-image persists. This is even though everyone they meet and know sings their praises and loves them for who they are. This could be despite months of mental health therapy, self-help, and consistent facts about themselves that contradict their low self-esteem, such as the ways they demonstrate that they are capable and virtuous in their day-to-day life:

“[….] I feel helpless in that fathers cannot protect their children; brothers cannot protect their sisters, and husbands cannot protect their wives. I do not know my health condition at all. You know this all happened in your homeland and your home, where everybody believes in feeling safe; this makes me nervous. I feel wrong about being a female and my incapability to protect myself from inhuman abuse (sobbing). Suppose I get ears to hear me. I wish to shout loud in a large audience or in public.” (Crying). (Source: a 30 year old married woman).

Psychological violence can include direct assaults on the body with objects or weapons, assault on girls, being denied access to women’s and girls’ homes, and deprivation of sleep or food. It is one of the types of violence committed in war-torn areas and sexual violence.

#### Threatened by weapons (bullet, knife, and bomb)

The perpetrators fired the bullet just near the victim’s body to intimidate the victim into accepting their order. They also intimidated the victims with knives, as they would slaughter them if they refused the rape or tried to escape. Sometimes the invaders intimidated the victims by showing them military armor (like a bomb) before practicing sexual violence:

“He fires a bullet and asks me to decide whether to go or die. I replied, as I was not interested in either of the choices. He then told me to turn around and give him my back to kill me. As soon as I turned around, my sister pleaded with me to accept the order and accompany them, assuring me that they would not harm me.” (Source: a 14 year old, 8th -grade student).

“[…] When he called me through the number that I gave him, that was not functional, and he hit me again. When I asked him not to hit me, he said, Why don’t you tell me the truth? and then he pulled out his knife and threatened, I am going to kill you, and no one will ask me.” (Source: a 18 year old, 11th -grade student).

##### Intimidation

The perpetrators have purposefully instilled psychological fear in the local population, particularly women and girls. They have used all available means to dehumanize women and girls verbally and physically. Survivors reported that perpetrators committed rape against women/girls in ways that violated societal norms, values, and traditions; they raped women in front of their children, making both women and their children feel ashamed, humiliated and intimidated.

“I am 35 years old and a wife of a Federal Police officer. I have two children. Because of the war, I was displaced for 18 days and stayed in a camp with no food to feed my children. Then, I decided to come back home to start selling coffee and tea to support my children. One day, two Tigrayan terrorist group members came to my home as they were informed that I am a wife of a Federal Police, and tried to search for weapons, but they found nothing. Sadly, they came back in the evening and raped me in front of my children; this inhuman act has left me in despair and depression. From that day on, I always felt ashamed of seeing my children. What they committed against me is a cruel and immoral act.” (Source: a 35 year old married woman).

“I came to Shoa Robit for Holy Water “Tsebel” with my sister. The invaders arrived on Saturday, November 20, 2021. They had spent the night in Shoa Robit. On Sunday morning, I felt sick and stayed inside, locking my room. Finally, they came to our dormitory and slammed our door. One of them targeted his gun on me. They accidentally found a military uniform in the house where we hid and humiliated my sister to tell them if her husband or any other is a soldier, and she told them that no one is a soldier. Then, they forcefully took me to another house and raped me there.” (Source: a 14 year old, 8th grader girl).

Other participated added.

“[….] The terrorist group came to the rural areas of Shoa Robit, where we fled to. A few minutes later, one carrying a big stick and a bag full of bombs intimidated my husband while another armed soldier targeted his gun on me to show him where the government has stored weapons which I have no idea about.”(Source: a 30 year old, 4th grader married woman, Part-time housemaid).

#### Interventions and support

Health professionals have provided psychological counseling in the districts of Showa Robit and Debre Birhan. The survivors reported the following regarding clinical care and support from the regional health.

They examined me for pregnancy. First, we came for a check-up, and then 15 days later, we were told to come back on the day of our appointment. We came and rechecked. I was told that I was not pregnant. I do not know if it was, but they gave me many pills, and I took them. They promised us to give support of about 3,000 birrs.

“There is no diagnosis and follow up of the victims to recover from the pain, and victims need mental support. There is stigma and discrimination in the community. Our willingness does not do it; there should be a moral support to all the victims in the community.”(Source: a 14 years old, 8th grader girl from the rural areas came to town for Holy Water treatment).

### Coping mechanisms

The research also solicited views from participants on issues relating to coping mechanisms after being subjected to rape. Rape is usually stigmatized, and the survivor is discriminated against. Being a victim of rape is embarrassing not only to the survivors but also for their families. Hence, the survivors were more concerned and fearful of the community reaction (whispers/gossip). Most survivors used different perceived coping strategies to avoid these community reactions. According to the informants, visiting health facilities, self-isolation, migration (living apart from the community/family), making a deliberate effort to forget the event, being silent, dropping out of school, and seeking advice from others were all used as coping mechanisms.

Survivors of GBV reported profound avoidance behaviors that reminded them of the trauma. For example they avoided activities such as fetching water from the local river and going to school. Some survivors migrated to other places where others did not recognize them and never returned to their residence. A 19-years-old married young woman in the tenth grade reported the following:

“[…] I usually worried and afraid of the village dwellers and did not go with anyone. […] I have not visited my family and lived in a rental property at Debark.” (Source: a 19 years old, married woman)

In the same way, another informant from North Showa zone reported that she and her friends decide to go to another place to lead their life and they did not want to go back to their hometown ever. This further complicated their future opportunities/life. This narrative implies that victims were forced to migrate to the distant area and face all the consequences following migration.

Some informants preferred to ‘keep silent as a perceived coping mechanism. The silence might be due to the cultural norm of fear of stigma. Sexual and reproductive issues are not topics of open discussion in the area. In general, such a topic is considered an embarrassing topic to openly talk about. Moreover, the community has conservative attitudes to make meaningful communication on this issue. However, being silent in case of rape may have lifelong consequences, as they may not get medical treatment, psychosocial and economic support.

However, few IDI narrated that experiencing rape is not the end of their life. Therefore, they decided to continue to pursue their education to help “*make their dreams come true*”. The survivors suggested that victims should be eligible for free health services, frequent psychosocial support, educational and economic support (job creation), and fair distribution of support (e.g., money). They acknowledge the government should work diligently to trace the GBV victims at the community level to decrease adverse reactions. One of the participants pointed out that:

“[…] It (rape) is not done by our willingness, […] there should be a moral support to all victims in the community.” (Source: a 32 year old, unmarried woman).

One of the challenges in the wake of invasion and war is its psychological and health consequences, particularly on vulnerable groups of the community such as women, children, elderly and people with disability. In this regard, raped women have suffered a great deal of fear, trauma, and stigma within the community on top of contracting Sexually Transmitted Infections. Fear of disclosure is another challenge raped survivors faced; this has arisen from the fact that the society will discriminate them and intimidate them for long.

“The survivors of GBV are not coming to the health center. After creating awareness in the community, four raped women came to our health facility for further investigation. We, thus, gave them some counseling and essential health support. We communicated with the specialist (gynecologist and obstetrician) for further diagnosis. In our catchment, 32 women have been raped by perpetuators. Some of them told us they are wives of priests and they do not want this information to reach their families (children and husbands). We have provided HIV screening, counseling and family planning services to these survivors.” (Source: a-27 year-old young Key Informant from North Wollo).

Other key informants reported the following:

“The Tigrean invading group has raped four women; some are raped at home while the remaining were exposed to rape while trying to flee to other places as the invading group controls their home area. We, as health professionals, have disclosed that rape survivors can get medical services from our health center; we expect that there will be several women and girls who experienced rape.” (Source: a 36 year old Key Informant from North Showa).

“It is difficult to quantify the exact number of raped girls/women in each woreda and Zones; yet I have seen three raped women in our catchment. I understand is that women and girls do not want to disclose the rape experience they faced. I believe we have to work hard to help these survivors.” (Source: a 29 year old Key Informant from North Wollo zone).

“I have met three raped girls who seek medical care at their homes. They manifest fear of stigma, pregnancy, catching HIV and other STIs. I anticipate that many other raped women and girls will come to our hospital for medication; thus, we need to be ready to give them due service. During the invasion, I observed parents’ worries about their daughters. Mothers were worried and stressed about how and where to hide their daughters from perpetrators.” (Source: a 41 year old Key Informant from South Wollo).

A bulk of data collected from survivors and key informants showed that perpetrators use immoral and normless acts against women and girls. This is purposely committed against women to show the level of revenge they have come up with. A pregnant woman needs support and respect in our society, but the invading group raped a pregnant woman, deviating from societal norms.

“The war brought about a damaging effect on the lives of women. The invading group committed morally offensive acts against women and girls. They have even raped an eight-month pregnant woman in Sekota which totally deviates from societal norms.” (Source: a 35 year old man Key Informant from Wag Hemra zone).

“The invading forces have raped two women, and we have sent them to D/Berhan hospital for medication. One of them has been identified as HIV positive. The informant narrated that the rapists have employed strategies to rape girls; for instance, one of the perpetrators came home and asked the family members that he needed a girl to cook him food. Instantly, a grand woman agreed to go with him and cook food, but he refused and even fired a bullet to calm her. In the meantime, they were terrified and kept silent; the perpetrator then forcefully took one of the girls to another house and raped her.” (Source: a 32 years old key informant from North Showa).

## Discussion

Here we report the study findings that document GBV among 1117 Ethiopians during the two years devastating war in Northern Ethiopia. The war broke out on November 4 of 2020 and ravaged the 3 contiguous regions of Amhara, Afar and Tigray until the war was concluded on November 3 of 2022, when an African Union brokered peace treaty led to cessation of hostilities and the armed rebel group agreed to disarm and demobilize its combatants. The detailed background to the armed conflict is beyond the scope of this study.

Sexual violence is common during times of war and national emergency, resulting in a decline in the observance of human rights, particularly for women and girls [4, 25, 26]. This is a fact among Amhara women and girls, indicating that sexual violence hinders or deprives women and girls’ ability to exercise their civil and political rights, economic, social, and cultural rights, and third-generation rights such as the right to peace and development.

Furthermore, post-conflict sexual violence is common. Post-conflict consequences of sexual violence include ongoing trauma, rejection by families and communities following their violence, unwanted pregnancies, stigmatization and ostracization of traumatised women, the spread of sexually transmitted infectious diseases and HIV, suicide and coerced suicide (under pressure from husbands or community members), and rape of women displaced by war and without male protection, including in refugee camps. Thus, the conflict and extremist fundamentalist aggression, as we see in the Amhara region war-affected areas, tended to breed more violence and negatively affected family relations. Therefore, in addition to so many other injustices on women’s bodies, the research exposed how the tragedy of war continues to play out in the future.

This research used In-Depth Interviews (IDI) of Gender Based Violence (GBV) victims and Key Informant Interviews (KII) of health professionals to determine that GBV was common, particularly among women who resided in the conflict-affected areas of Northern Ethiopia. It showed that participants were exposed to various types of GBV and its consequences. The study also revealed that the armed conflict had several health consequences including breakdown of health and social services and heightened risk of disease transmission. Furthermore, in line with other studies [4, 6, 8], our findings showed that the armed conflict was associated with an increasing frequency of GBV, especially against women. However, GBV was underreported [27] and establishing the exact rates of GBV was challenging [28] in northern Ethiopia. Thus, community based-study should be conducted to estimate the degree of GBV in war affected.

In this study, both (male and female) sexes were reported as survivors of GBV in war-affected areas, which is consistent with other studies [6, 29]. Although reliable data using community-based studies are needed to quantify the GBV survivors of armed conflict, many GBV survivors reported seeking healthcare services in our study after armed combatants left the invaded zones. In our study, all of the registered GBV survivors (n = 1177) had faced violence by the invading perpetrators. The proportion of reported GBV survivors varied between Zones, which could be attributed to the variation in the duration of stay of the invading Tigrayan forces in different areas. Moreover, this study was carried out in the midst of the armed conflict therefore, the proportion of the GBV victims is probably a gross underestimation of the actual number of GBV victims. For example, the perpetrators, i.e. Tigrayan invading forces, occupied a relatively long period (six months) in North Wollo (where the highest proportion of GBV survivors were reported) compared to the Oromo special zone (one month). Therefore, each zone should work together with other stakeholders to identify GBV survivor including males and provide rehabilitation services.

The qualitative data acquired from study participants also reported that invading Tigrayan forces did gang rape and other forms of coitus forcefully in front of their children and husband, which is in line with the findings in South Sudan [26]. Studies showed that sexual violence could have multiple consequences for survivors, including social impacts and adverse health outcomes [12, 25, 30]. Social impacts and health consequences of sexual violence such as rejection from family and community, dropping out of schools and jobs, unwanted pregnancy, gynecological problems (vaginal discharging), and acquiring sexually transmitted infections were documented in our qualitative findings, which are consistent with the findings of numerous other studies [12, 30–32]. Regular counseling, support and health checkup e.g. HIV and other health problems should be given for GBV survival.

GBV survivors in our study experienced multiple and compounding forms of violence (sexual, physical, and psychological violence) during times of invasions by armed combatants. In this study, GBV survivors responded that they had experienced physical violence; slapping, burning the body with cigarette fire, hanging the neck and being threatened by weapons (bullet, knife and bomb), which align with the findings of research undertaken in both Côte d’Ivoire and South Sudan [6–8, 26]. In line with other studies, the possible causes of GBV in our study area could be the use of GBV as a low-cost “weapon of war” to achieve strategic goals by perpetrators, such as clearing a civilian population from a specific area. In this case, it is “military committed to instill terror in a population” (Bastick, Grimm and Kunz: 14); in other cases, it is part of genocide, contributing to efforts to annihilate a specific ethnic group [29, 33].

Similarly, because women in developing countries are frequently in charge of basic economic support activities essential to their families’ daily lives, violence against them is used as a weapon of social disruption [34, 35]. Additionally, it could be related to the interest of the perpetrators to use sexual violence to punish or humiliate a perceived enemy group. This implies that violence based on gender is regarded as the most heinous form of cruelty, severely impeding an individual’s ability to enjoy rights and freedoms and seriously jeopardising the transition from armed conflict to peace. Furthermore, it profoundly impacts women, girls, men, and boys worldwide during armed conflicts and long after they have ended. As a result, gender-based violence undermines the connection between security and development. GBV is widely regarded as the ultimate generational form of violence. In line with other studies [25, 26], this study showed that GBV has many adverse health effects among survivors and further exacerbates GBV survivors’ families’ lives.

Post-conflict consequences of sexual violence include ongoing trauma, rejection by families and communities following their violence, unwanted pregnancies, stigmatisation and ostracisation of traumatised women, the spread of STIs and HIV, suicide and coerced suicide (under pressure from husbands or community members), and rape of women displaced by war and without male protection, including in refugee camps [26]. Thus, the conflict and aggression, as we see in the Amhara region war-affected areas, tend to breed more violence and negatively affects family relations so that the tragedy of war plays out, like so many other injustices on women’s bodies in the future. In addition, this study reported stigma and discrimination, suicide attempts, nightmares, post-traumatic stress disorder, depression, and hopelessness in survivors of gender-based violence. The findings are consistent with an earlier study [18, 36, 37]. Systematic reviews in low-and middle-income countries showed that post-traumatic stress disorder and mental disorders were documented among GBV survivors in war-affected areas [38–41]. Many people suffer from mental health issues resulting from sexual and other forms of gender-based violence; it is critical to improve the health and well-being of survivors and prevent further ill health and promote well-being. Despite the devastating and alarming effect of the war on both sexes [community] in the northern parts of Ethiopia, the participants in our study reported outmigration (leaving their residency), visiting health facilities, self-isolation, deliberately forgetting the event, being silent, school drop-out, and getting advice from others as a means of coping. Though coping describes how GBV survivors detect, appraise, deal with, and learn from stressful encounters, using the aforementioned coping strategies in war-affected areas exacerbates the problem. As a result, this study suggests that survivors of GBV in northern Ethiopia seek counselling and strengthen the community-based support systems. Furthermore, more focused and intensive research efforts should be undertaken to isolate the effects of specific strategies to improve well-being and prevent or treat mental disorders in order to strengthen the knowledge base of effective practices for the regrettably large population of survivors of sexual and other forms of gender-based violence in the context of armed conflict.

### Strengths and limitations

While this study substantially contributes to the international academic literature on sexual violence in war-torn areas, some limitations should be recognized. This study was conducted in Amhara Region. For this reason, it does not reflect the sexual violence experiences in other war-torn areas such as the Afar region, which was invaded by Tigrayan invading forces in three separate armed insurgency campaigns in October 2021, December 2021 and March 2021. It also does not include sexual violence in the Tigray region. Further, study participants were self-referred to healthcare facilities or identified using a snowball sampling approach. These nonrandom approaches may lead to bias and lack of representation. Also, given the extent of the conflict, the displacement, and the continuing conflict, this research study may be limited to estimate the degree of GBV during the armed conflict. This is in part due to shame and guilt associated with GBV thus not may many victims are likely to report the trauma. Future systematic, population-based studies are needed to address these limitations.

## Conclusions

GBV survivors experience multiple and compounding forms of violence (sexual, physical, and psychological violence), which has a myriad of adverse health effects among survivors and their families. Outmigration, visiting healthcare facilities, self-isolation, making a deliberate effort to forget the traumatic event, being silent, school drop-out, and getting advice from others were perceived coping mechanisms by the victims. Thus, GBV survivors urgently need multidimensional intervention programs rooted on trauma-informed care such as psychological support, healthcare support and follow-up and economic empowerment to lead their everyday life in the community. Given the long-lasting impacts of trauma, these intervention programs need to be long-lasting. In addition to the HIV/AIDS stigmatization also suffered by many individuals in these communities creates a ‘dual’ stigmatization that requires significant support and awareness at the community level. Further research is needed to generate the extent of GBV, mental health outcomes, and other health effects of victims of physical, psychological, and sexual violence in conflict-affected settings in order to help understand the magnitude of the problem and identify potential solutions to address it.

In summary, additional community-based study is required to determine the magnitude of GBV survivors in war-affected areas for resource allocation and prioritization, provide need-based psychosocial support, and meet the needs of armed conflict-related GBV survivors.

## Data Availability

Due to the sensitive nature of some of the interviews, the qualitative data was not publicly available. Additional information may be obtained from the first author or corresponding author.

## List of abbreviations

GBV: Gender-based violence
HIV: Human Immune Deficiency Virus
IDI: In-depth interview
KII: Key Informant Interviews
STIs: Sexually Transmitted Infections
UN: United Nations
WHO: World Health Organization
