# Correction to: Gender-based violence in the context of armed conflict in the Amhara Region, Northern Ethiopia

**DOI:** 10.1186/s13031-025-00665-1

**Published:** 2025-04-28

**Authors:** Desalew Salew Tewabe, Muluken Azage, Gizachew Yismaw Wubetu, Sisay Awoke Fenta, Mulugeta Dile Worke, Amanu Mekonen Asres, Wallelign Alemnew Getnet, Genet Gedamu Kassie, Yonatan Menber, Alemtsehay Mekonnen Munea, Taye Zeru, Selamawit Alemayehu Bekele, Sadiya Osman Abdulahi, Tigist Biru Adamne, Hiwot Debebe Belete, Belay Bezabih Beyene, Melkamu Abte, Tesfaye B Mersha, Abel Fekadu Dadi, Daniel A Enquobahrie, Souci M. Frissa, Yonas E. Geda

**Affiliations:** 1 Amhara Public Health Institute, Amhara Region, Bahir Dar, Ethiopia; 2 School of Public Health, College of Medicine and Health Sciences, Bahir Dar University, P.o.Box: 79, Bahir Dar, Ethiopia; 3 Emergency Response and Recovery Officer, Amhara Region, Bahir Dar, Ethiopia; 4 Department of Midwifery, College of Health Sciences, Debre Tabor University, Debre Tabor, Ethiopia; 5 Faculty of Social Science, Bahir Dar University, Bahir Dar, Ethiopia; 6 Institute of Public Health, College of Medicine and Health Sciences, University of Gondar, Gondar, Ethiopia; 7 Bureau of Women Children and Social Affairs, Amhara Region, Bahir Dar, Ethiopia; 8 Amhara Regional Health Bureau, Amhara Region, Bahir Dar, Ethiopia; 9 Cincinnati Children’s Hospital Medical Center, University of Cincinnati, Cincinnati, OH USA; 10 Menzies School of Health Research, Charles Darwin University, Casuarina, Australia; 11 Department of Epidemiology, School of Public Health, University of Washington, Seattle, WA USA; 12 Institute of Psychiatry, Psychology and Neuroscience, Health Service and Population Research Department, Centre for Global Mental Health, King’s College London, London, UK; 13 Department of Neurology and the Franke Barrow Global Neuroscience Education Center, Barrow Neurological Institute, Phoenix, AZ USA

**Correction to: Conflict and Health (2024) 18:1**

10.1186/s13031-023-00563-4

In this article the title was incorrectly given as ‘Gender-based violence in the context of armed conflict in Northern Ethiopia’ but should have been ‘Gender-based violence in the context of armed conflict in the Amhara Region, Northern Ethiopia’.

• In Background section, in the sentence beginning ‘The war took place in the Amhara,…’ in this article, the text **“**The current study was carried out by the Amhara Public Health Institute and Bahir Dar University in the Amhara region**”** should have read ”The current study was carried out by the Amhara Public Health Institute and Bahir Dar University in Amhara Region, northern Ethiopia. At the time of the study, we did not have access to Afar and Tigray Regions of northern Ethiopia.”

• In paragraph 5, line 17 to 19,… the text “Therefore, this study aims to explore lived experiences, and consequences of survivors of GBV in war-affected areas of northern Ethiopia should have read “Therefore, this study aims to explore lived experiences, and consequences of survivors of gender-based violence in war-affected areas of the Amhara Region, northern Ethiopia.”

In discussion section, the text “Here we report the study findings that document GBV among 1117 Ethiopians during the two years devastating war in Northern Ethiopia.” Should have read “Here we report the study findings that document GBV among 1117 Ethiopians during the two years devastating war in the Amhara Region, northern Ethiopia.”

• In paragraph 4, line 5, the text, “This research used In-Depth Interviews (IDI) of Gender Based Violence (GBV) victims and Key Informant Interviews (KII) of health professionals to determine that GBV was common, particularly among women who resided in the conflict-affected areas of Northern Ethiopia.” Should have read

This research used In-Depth Interviews (IDI) of Gender Based Violence (GBV) victims and Key Informant Interviews (KII) of health professionals to determine that GBV was common, particularly among women who resided in the conflict-affected areas of the Amhara Region, northern Ethiopia.

• In paragraph 4, line 9. the text “However, GBV was underreported [27] and establishing the exact rates of GBV was challenging [28] in northern Ethiopia” should have read “However, GBV was underreported [27] and establishing the exact rates of GBV was challenging [28] in Amhara Region, northern Ethiopia.”

• In paragraph 5, line 7, The text “Although reliable data using community-based studies are needed to quantify the GBV survivors of armed conflict, many GBV survivors reported seeking healthcare services in our study after armed combatants left the invaded zones.” Should have read, “Although reliable data using community-based studies are needed to quantify the GBV survivors of armed conflict, many GBV survivors reported seeking healthcare services in our study after armed combatants left the invaded zones of Amhara Region, northern Ethiopia”.

• In Strengths and limitations section, the text “This study was conducted in Amhara Region” should have read, “This study was conducted in the Amhara Region only, but not in the Afar or Tigray Regions, northern Ethiopia.”

• In Strengths and limitations section, the text “These nonrandom approaches may lead to bias and lack of representation” should have read “The nonrandom approaches to participant identification and the fact that the study was limited to Amhara region and did not include Afar and Tigray regions of northern Ethiopia; may lead to bias and lack of representation of inhabitants of the conflict areas in northern Ethiopia.”.

